# Entrepreneurial networks, geographical proximity, and their relationship to firm growth: a study of 241 small high-tech firms

**DOI:** 10.1007/s10961-022-09988-0

**Published:** 2022-12-25

**Authors:** Hans Löfsten, Anders Isaksson, Heikki Rannikko

**Affiliations:** 1 Chalmers University of Technology, 412 96 Göteborg, Sweden; 2 Chalmers University of Technology Metropolia University of Applied Science, Vanhamyllynranta 13, 01840 Kaukkala, Finland

**Keywords:** Entrepreneurial networks, Geographical proximity, Firm growth, New technology-based firms, L26, M13, O30, O32

## Abstract

Start-up firms in high-tech sectors normally engage in networking to overcome their lack of resources, knowledge, and competence constraints. A newly established firm’s network can provide a source of social capital, which may enhance its growth prospects. In this study, 241 new technology-based firms (NTBFs) in Sweden are studied during their early formative years to investigate how entrepreneurial networks and the geographical proximity to actors in these networks affect the early performance of these firms in terms of growth. Three underlying factors are identified in the analysis: geographical proximity and professional and consultative networks. This study finds that professional networks have a positive and significant effect on NTBFs’ growth, which indicate that utilizing these networks benefit the growth of both young and growing firms. NTBFs in initial stages can acquire business opportunities by constructing professional networks. In addition, several formal links positively affect growth, such as regional business partners, incubator networks, and links to universities.

## Introduction

Networks can facilitate acquisition of knowledge (Song et al., 2017), including market and technological knowledge endowments, and the utilization of entrepreneurial and market opportunities (Gruber et al., 2008, 2013; Wiklund & Shepherd, 2003). The concept of an entrepreneurial network is generally associated with entrepreneurs who are organized either formally or informally to increase small and new firms’ levels of business activities (Das & Teng, 1997). An entrepreneur’s network, which is considered a source of social capital, can increase firm performance (Cho & Lee, 2018; Smith and Lorke, 2008). Rubino and Vitolla (2018) show that network characteristics influence firms’ performances differently because of network diversity and geographical openness. Romijn and Albu (2002) explore how innovative performance of new high-tech firms are related to their external networking activities and depend on the firms’ geographical proximity in these networks. They find that the regional science base plays an important role in supporting new technology-based firms (NTBFs).

Small high-tech firms play an important role in firm growth, reduce unemployment, and promote innovation in an economy (Storey, 1983). Over the years, NTBFs have rapidly grown as has the economy in areas with high NTBF concentrations. In addition, policy makers and researchers increasingly focus on stimulating the growth of NTBFs (Bager-Sjögren et al., 2017). Emerging NTBFs encounter two major obstacles, namely a lack of resources and information uncertainty. They may overcome these obstacles by participating in entrepreneurial networks where they can receive information, knowledge, and exchange resources.

Several studies examine innovation in technology network structures in terms of sharing technology projects and agreements (Hargadon & Sutton, 1997; Tiwana, 2008). Research and development networks are crucial for NTBFs’ to be innovative (Börjesson & Löfsten, 2012; Löfsten, 2015). Nikiforou et al. (2020) find that market network structural holes are positively associated with firms’ entrepreneurial behaviors. In addition, technological knowledge networks also increase entrepreneurial behavior if an open market network is established. Market networks can adequately overcome newness and smallness and improve a firm’s performance (Ostgaard & Birley, 1994; Lechner et al., 2006).

Durda et al. (2019) analyze ties in the establishment and development of start-up firms, where the key actors are business partners and friends and angel investors who provide business insights, technology, and marketing advice. Park et al. (2020) stated that managing competition uses much of an entrepreneur’s time—time that is instead required to deal with other critical activities. Brown et al. (2019) claim that spatial proximity is important in pre-crowdfunding networks where incubator or accelerator programs are critical. Parida et al. (2016) claim that NTBFs normally engage in networking to overcome constraints in innovation-based competition. Svare et al. (2020) show how trust based on perceived benevolence, ability, and integrity influences different dimensions of innovation network outcomes. However, participation within the network is not without constraints. The network structure can change over time which changes what the entrepreneur can obtain from the network.

Entrepreneurial networks are strategic tools in achieving firm performance, and several scholars have studied the importance of networking, connections, and firm success (Abu-Rumman et al., 2020; Baum et al., 2000; Chu & Yoon, 2021; Daneil, 2010; Kalm, 2012; Karami & Tang, 2019; Madzimure, 2019; Raza et al., 2018; Rubino & Vitolla, 2018; Wang & Fang, 2021). The importance of entrepreneurial start-ups’ resources regarding networks is also recognized (Coviello, 2006; Hite & Hesterly, 2001). A critical analysis of earlier studies indicates a gap in the literature because many of these studies disregard very small (less than three employees) and young (less than 3 years old) firms in their analyses of the high-tech sector, entrepreneurial networks, and geographical proximity and its relation to growth. The geographical proximity between firms and other organizations is an important factor affecting their abilities to develop networks. Sandberg and Alvesson (2011, p. 23) refer to the process of finding gaps in the literature as “gap-spotting.” Based on the background presented above, this study aims to explore the effect of entrepreneurial networks and geographical proximity on early growth of NTBFs. In particular, (1) this study considers that entrepreneurial networks comprise formal relationships between NTBFs and the environment in terms of geographical proximity to different actors and attractive or expansive industrial areas; (2) therefore, this study analyzes firms’ entrepreneurial networks and geographical proximity in relation to business performance, specifically early growth.

It contributes to the literature by exploring how very small NTBFs can use resources, such as entrepreneurial networks and proximity, to achieve early firm growth. Proximity is identified as one of the conditions for developing a network structure and is considered in this study as a geographical effect, that is, geographical proximity. Proximity can explain why NTBFs choose locations that are near a university, firms in the same industry, or customers or that are in an incubator. In addition, it can explain why geographical proximity between organizations can be crucial for their ability to develop entrepreneurial networks and generate the necessary resources for firms’ abilities to perform.

This study determines which types of entrepreneurial networks are the most beneficial for NTBFs and identifies which contingencies (e.g., geographical proximity) benefit these networks the most. From the perspective of NTBFs in their early start-up phase, human capital, or the resources related to the founder, and external relations are important for the firm’s performance. This study has implications for business actions and hence firm policies, as the knowledge on how entrepreneurial networks and geographical proximity provide valuable insights for founders and managers. To analyze the relationships between the activity levels in entrepreneurial networks, geographical proximity, and early firm growth, the following research question is formulated:

**RQ:***Do entrepreneurial networks and geographical proximity explain NTBFs early growth* *variance?*

This study measures the impact of entrepreneurial networks and geographical proximity on the growth of small high-tech firms. A questionnaire was administered to 241 NTBFs in Sweden on entrepreneurial networks and geographical proximity. The selected firms were very small with approximately two employees. The rest of the paper is structured as follows: Sect.  2 provides an overview of the network and proximity literature. Section 3 describes the sample, method, and variables. Section 4 presents the statistical analysis and discussion of empirical findings, and Sect.  5 presents the conclusions.

## Literature review and hypotheses

### Entrepreneurial networks

Over the years, scholars have increasingly examined how the social contexts in which firms operate influence their behaviors, stimulate innovation and performance, and increase the firms’ competitiveness (Ahuja, 2000; Davidsson & Klofsten, 2003; Cantner et al., 2010; Gulati et al., 2000; Fonfara et al., 2021; Jiang et al., 2018; Huggins & Thompson, 2016; Raskovic et al., 2012; Rydehell et al., 2019). Networks are defined as sets of individuals or organizations that interact with each other, and these interaction networks are inter-connected (Greve, 1995; Håkansson et al., 2009; Ostgaard & Birley, 1994). Entrepreneurs can also use network resources to evaluate new technologies, for marketing opportunities and to acquire legitimacy (Pettersen & Tobiassen, 2012; Sullivan & Marvel, 2011). Strategic networks, such as those formed by small firms, are important when analyzing entrepreneurial ventures (Gulati et al., 2000; Stuart & Sorenson, 2007). Surangi (2013) refers to the important network resources for an entrepreneur: information; access to finance; access to skills, knowledge, and advice; social legitimacy; and reputation and credibility. Furthermore, empirical findings emphasize the centrality of networks in entrepreneurial processes.

Entrepreneurial networks help NTBFs attain knowledge, technology, and social resources and improve their performance (Aldrich & Zimmer, 1986; Stuart and Sorensen, 2007; Partanen, 2020; Weizhen et al., 2022). Studies on entrepreneurship have examined network structures and performance (Hite & Hesterly, 2001; Hoang and Antonic, 2003). Similar to this study, some studies have also examined the networks, business performance, and their interrelationships (Acquah Obeng, 2019; Baker, 1998; Burlina, 2019; Chakravarty et al., 2020; Goerzen et al., 2005; Gronum et al., 2012; Karami & Tang, 2019; Naude et al., 2014; Podolny, 1993; Rubino & Vitolla, 2018; Watson, 2007; Wang & Fang, 2021; Zacca et al., 2015). This research shows that access to good networks may have economic benefits. Scholars identify two fundamental dimensions of entrepreneurial networking (Surangi, 2013): (i) structural and (ii) relational. The structural dimension relates to the pattern of relationships between actors (Klyver et al., 2007).

Social networks may provide entrepreneurs with the necessary resources and boost their firms’ business performances (Hansen, 1995; Jensen, 2001; Ripolles & Blesa, 2005; Wang & Schøtt, 2021). In addition, social networks can affect entrepreneurial intention (Hmieleski & Corbett, 2006) and orientation (Ripolles & Blesa, 2005). Entrepreneurial networks are also important for identifying entrepreneurial opportunities (Yu et al., 2021). Entrepreneurs struggle to commercialize their products when they do not fully understand the markets (Adams et al., 2019). Generally, an entrepreneurial opportunity is a key factor that determines an NTBF’s performance (Davidsson, 2015; Foss et al., 2013). Studies show that new business opportunities are attributable to business networks (Aldrich & Cliff, 2003; Cantù, 2018; Tamasy, 2006). The quality of entrepreneurial networks is also important, and researchers discuss if the quality of networks facilitates identifying opportunities (Cardon et al., 2017; Hoang & Antoncic, 2003). Panda (2014) finds that managerial networking is a significant determinant of firm growth.

The general assumption is that informal links are more prominent in the earlier stages, and they help to overcome challenges concerning resource access and limited awareness of resources and opportunities (Delmar & Shane, 2003; Hite & Hesterly, 2001). New and expanding firms are less likely to be aware of the entire range of opportunities and threats because of their limited search abilities. Therefore, they most likely rely on informal ties (Birley, 1985; Hoang & Antoncic, 2003). Thus, informal networks are likely to be created at the earlier stages of a firm’s business cycle. Anderson and Medlin (2016) focus on the temporal and dynamic ways in which actors seek value through business opportunities. In addition, there are two different perspectives of networks in the related literature: closure and openness, that is, the structural hole perspective (Nikiforou et al., 2020). Structural holes give a firm access to unique information (Burt, 2004).

To summarize, for high-tech start-ups to build a good entrepreneurial network it is necessary to identify the network characteristics that matches the NTBF. This study focuses on industrial sectors where the business environment is dynamic and uncertainty is high, which necessitates information gathering. Therefore, entrepreneurial networks are important in entrepreneurship. Accordingly, the first hypothesis is:

**H1**: Entrepreneurial networks positively influence early growth for very small and young high-tech firms

### Geographical proximity

Geographical proximity, meaning the extent to which key actors and resources are located geographically close to each other, is an important condition for networks (Soh, 2003; Walker et al., 1997). With geographical proximity, networks can become a resource, because the NTBF can obtain access to advice, capital, and innovation (Gulati, 1999; Santamaría et al., 2021; Zhang et al., 2022). The resource-based theory focuses on performance and also on intangible concepts, such as networks and proximity, which offer an opportunity to focus on technology, innovation, and business performance. Clusters, innovation systems, technology districts, and science parks are examples of areas where the issue of proximity has grown in importance in the analysis of economic environments, and firm performance. Studies analyze proximity relations (Torre and Gilly, 1999) and especially focus on innovation (Baptista & Mendonça, 2009; Gallie, 2009). Hence, most of the research about different dimensions of proximity is conducted in the area of entrepreneurship.

Scholars also discuss another effect of proximity—it can be harmful (Boschma, 2005, p. 66) and lead to lock-ins. For example, too much organizational proximity may impact knowledge transfers negatively (Balland et al., 2015; Boschma, 2005; Johnston & Huggins, 2016). The same can be said of social proximity (Uzzi, 1996). These two-fold effects are referred to as the proximity paradox (Huber, 2012; Parjanen & Hyypiä, 2018). Bignami et al. (2020) find that collaborations in core knowledge areas are more negatively affected by geographical distance than collaborations within knowledge exploration areas. Geographical proximity is important, because neighborhood effects impact institutional and cultural settings (Hansen, 2015). Boschma and Frenken (2010) claim that proximity is central when studying networks and different actors regardless of their location. However, Boschma (2005) categorizes proximity as cognitive, organizational, social, institutional, and geographical, which facilitates analyzing actors’ dynamics in specific locations.

Geographical proximity, when actors seek to narrow the distance between them, can be developed in a certain area by the creation of localized innovation clusters through the development of local networks. Scholars have examined the effect of geographical proximity on firm performance from two perspectives. The first considers proximity to be the same as competition (Kalnins, 2004; Pancras et al., 2012), and the second focuses on clustering (Lu & Wedig, 2013; Tracey et al., 2014). In the first scenario, researchers focus on competition as a function of distance and claim that competing firms located close to each other may compete for the same customers and resources, which might result in cannibalization (Kalnins, 2004). However, cannibalization decreases as the distance between organizations increase (Pancras et al., 2012).

In addition, scholars seeking to assess the importance of the different types of proximity in firms’ performance often confirm that geographical proximity cannot ensure high performance by itself. For instance, Feser et al. (2008) found no connection between employment growth and technology-based clusters. McDonald et al. (2007) found that although UK clusters are linked to employment growth, clusters with deep collaborative networks are not. Spencer et al. (2010) claim that firms in industries located in an urban region with a critical mass of related industries, tend to generate both higher incomes and rates of employment. Younger firms tend to benefit more from agglomeration according to studies on the clustering of new firms (Feser et al., 2008; Gilbert et al., 2008; McCann and Folta, 2011). Gilbert et al. (2008) systematically analyzes different geographical proximity types and finds that new ventures located in clusters both absorb more knowledge from the environment and have higher growth and innovation. In summary, in line with the conceptual arguments and previous empirical findings, the following hypothesis is proposed:

**H2**: Geographical proximity positively influences early growth for very small and young high-tech firms

**Fig. 1:**
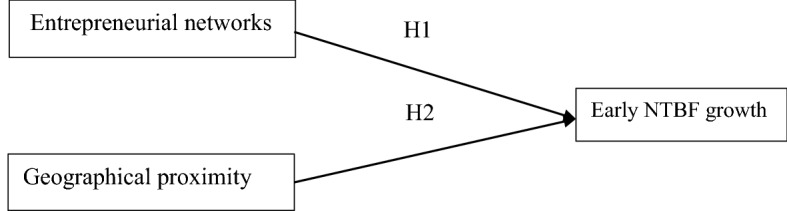
Conceptual framework

The conceptual framework is presented in Fig. 1. To capture this conceptual framework, including Hypotheses 1 and 2, entrepreneurial networks are measured according to the importance of the NTBFs’ links to different actors in entrepreneurial networks, such as accountants, banking institutions, consultants, and regional business partners. Geographical proximity measures the importance of NTBFs’ proximity to universities, customers, competitors, and large, well-known firms. Early firm growth is measured during the initial 3 years from 2014 to 2016. This study recognizes the complex nature of networks as resources, and the conceptual model explains how network structures and geographical proximity affects early NTBF performance.

## Methods and data

### Sample and data collection

This study focused on Swedish NTBFs that were registered in 2013 with their first full accounting year ending in 2014. The Retriever Business database (https://www.retrievergroup.com/) was used to find a suitable sample and collect business performance (growth: secondary business data). The Retriever Business database includes business information on all Swedish firms regardless of their formations or sizes. However, for our sample we limited NTBFs to independent firms that were incorporated as limited companies. This study focused on moderately high and high technology and knowledge-intensive firms. The NTBFs were aggregated at the two-digit level to guarantee anonymity. To ensure that the NTBFs had started trading, the sample was controlled in terms of value added tax and tax prepayments and all the firms were registered in 2013. In addition, it was vital that the NTBFs had updated contact information to ensure that answers were received from active firms.

The sample consisted of 1290 firms that were founded in 2013. To ensure further sample validity, the questionnaires were administered telephonically by TNS-Sifo (National Institute for Consumer Research), which is a professional marketing research firm in Sweden. Acceptable responses were received from 241 firms (response rate of 18.7 percent). Table 1 shows the characteristics of NTBFs in comparison to non-respondents. The firms that had not responded had fewer employees, sales, and assets; this indicates that many of them might not have been operational.

**Table 1:** Descriptive statistics for the surveyed 241 new technology-based firms started in 2013, Sweden

	Sweden
*Sample and response rate (number of firms)*	
N (population)	1290
n (response)	241
No response	1049
Response rate (%)	18,7%

		Sweden
*Non-response analysis based on the difference between respondents and non-respondents*		
*Accounting data for sampling year of 2014*		
Employees^a^	Respondents	1.95
	Non-respondents	1.24
	p value	0.391(n.s.)
Sales^b^	Response	240.37
	Non-respondents	160.53
	p value	0.620 (n.s.)
Assets^b^	Response	152.20
	Non-respondents	89.28
	p value	0.674 (n.s.)

NACE revision with two codes	Number of firms	Percent
*Industry sector and innovation performance (responding firms), year 2016*		
High-tech manufacturing	15	4.5
Medium high-tech manufacturing	21	8.5
High-tech Knowledge intensive	205	87.0
Sum	241	100.0

Innovation performance—patent^c^	Mean	Std
High-tech manufacturin	0.47	1.13
Medium high-tech manufacturing	0.24	0.63
High-tech Knowledge intensive	0.12	1.00

^a^Number of employees

^b^1000 Euro

^c^Number

The respondent firms had few employees (mean 1.95, year 2014). The Swedish Standard Industrial Classification (SNI) is based on the EU recommended standard NACE Rev. 2 (Statistics Sweden) (activity classification). Table A in the Appendix shows the two-level SNI-codes and production units. NTBFs are classified according to their activities and a firm can have several activities (SNI-codes). The largest group in the sample (computer programming, consultancy, and related activities) accounted for 58.1 percent of the responses.

The survey was conducted in several steps. First, a pilot study was conducted in which our measures were developed from interviews with 26 NTBFs. All the study participants provided informed consent. After the questionnaire was developed, it was also tested on a smaller group of NTBFs. TNS-Sifo conducted the final survey in March and April 2016, telephonically. Reliability was ensured by implementing several controls in the data collection process. The questions in the survey were on firms’ initial conditions, regarding entrepreneurial networks and proximity. All measures were based on a 5-point Likert scale.

The sample was restricted to firms that had valid telephone numbers so that interviewers could contact them. Certain firms could not be located or had no operational activity and were classified as non-respondents. NTBFs were also classified as non-respondents if they chose not to participate due to time constraints, could not be reached due to incorrect contact details or answering machines, and because it was against the firm’s policy (see Table 2). The interviewers from TNS-Sifo are trained to conduct the survey, and reliability was further increased by using selected experienced callers. To further ensure response quality, the interview process was recorded and monitored. Usually, very small high-tech firms have only one person in a managerial position.

**Table 2:** Different reasons for non-response (frequencies)

Reasons for non-response	(%)
Could not get hold of the respondent	50.5
Didn’t want to participate	30.8.
Answers	18.7
Total	100.0
*Could not get hold of the respondent*	
Answering machine, occupied	64.6
Wrong number, reference tone	18.9
Away	2.7
No answer (15 contact attempts)	2.5
Do not speak Swedish	2.0
At another workplace	0.9
Other	8.4
Total	100.0
*Didn´t want to participate*	
Refusal—no time, do not want	79.2
Came back later	13.5
Refusal—principle, policy	7.3
Total	100.0

### Variables

#### Dependent variable: Growth 2014 to 2016

In the entrepreneurship research field, three business performance measures are suitable: profit, sales growth, and employment growth (Barkham et al., 1996; Davidsson, 1989; Delmar, 1996; Hoy et al., 1992; Zahra, 1991). Business performance was measured using the log-difference of sales (ln [Sales2016] – ln [Sales2014]) as suggested by Törnqvist et al. (1985). One problem with young firms is the highly skewed nature of measures of firm performance (Almus & Nerlinger, 1999; Coad et al., 2014; Delmar et al., 2013; Törnqvist et al., 1985), and using the log-difference of sales offers monotonic transformations that do not affect the ranking of firms. We used sales in 2014 as the starting point because it is the first full accounting year after a firm was registered and 2016 as the end year to capture the growth as close as possible to the time when the survey data were collected.

#### Independent variables

*Entrepreneurial networks* Formal networks include suppliers of capital, and professionals (Löfsten & Lindelöf, 2003; Löfsten, 2016b). An NTBF can acquire legitimacy by joining or cooperating with such organizations even before the firm has gained enough traction to become competitive. Associations of this formal type is a useful source of information that may impact areas of a business such as technical developments, communication, or administration. The formal networks can be regarded as open, according to the structural hole perspective, because the firm maintains relationships which are not connected to each other (Burt, 2004). Some of the associations relate to the general operations of all firms, such as accountants, banking institutions, chamber of commerce, consultants, lawyers, and regional business partners. However, we consider these services to be essential for NTBFs, especially patent advisers, venture capital firms, and incubator networks. In this study, only formal networks were used in the analysis. Building upon measures of business networks (Löfsten & Lindelöf, 2003; Rydehell et al., 2018), respondents were asked, “To what extent have the following actors affected the development of your company?” The applicable actors were accountants, banking institutions, chambers of commerce, consultants, lawyers, regional business partners, patent advisers, venture capital firms, and incubator networks.

*Geographical proximity* Geographical proximity, such as closeness to actors (Maine et al., 2010), provide firms with different resources for learning and knowledge (Löfsten, 2016a). Proximity between NTBFs and universities and research institutes also support an exchange of ideas and knowledge (Deeds et al., 2000). Balconi et al. (2004) claim that proximity supports networking and technology transfers (Niosi, 2006a, 2006b). Geographical proximity is a proximity category where different actors seek to get closer to certain people, places, or organizations. In addition, it can be enhanced in certain areas by creating localized innovation clusters to exchange knowledge (Boschma, 2005; Kalnins, 2004; Lu & Wedig, 2013; Pancras et al., 2012; Rydehell et al., 2019; Torre, 2008; Tracey et al., 2014).

All the measures were based on 5-point Likert scales to capture the extent of NTBF activities, specifically regarding how firms value entrepreneurial networks and geographical proximity. The question regarding geographical proximity was: “To what extent have the following dimensions affected the localization and development of your company?” Overall, the 16 variables (see Table 3) measure the variety of sources for advice and proximity, which are considered important for NTBFs.

**Table 3:** Descriptive statistics of the variables used in the study (*n* = 241)

	Mean	Std	Scale
*Entrepreneurial networks*			
1. Accountants	1.51	0.97	1–5
2. Banking institutions	1.49	0.90	1–5
3. Chamber of commerce	1.12	0.53	1–5
4. Consultants	1.85	1.21	1–5
5. Lawyers	1.41	0.84	1–5
6. Regional business partner	1.59	1.19	1–5
7. Patent advisers	1.22	0.72	1–5
8. Venture capital firms	1.18	0.70	1–5
9. Incubator network	1.50	1.08	1–5
*Geographical proximity*			
10. Proximity to university	1.65	1.24	1–5
11. Proximity to customers	2.74	1.54	1–5
12. Proximity to competitors in the same field	1.48	0.94	1–5
13. Local/regional advantages	1.80	1.22	1–5
14. Attractive/expansive industrial area	2.28	1.42	1–5
15. Proximity to large, well-known firms	2.14	1.46	1–5
16. Proximity to similar firms	1.78	1.14	1–5
*Control variables*			
17. Product or service	4.01	1.30	1–5
18. Product/service differentiation	3.00	1.25	1–5
19. Pricing	2.81	0.91	1–5
20. Product/service quality	3.91	.080	1–5

#### Control variables

To distinguish the growth from the design of a firm’s products and services, control variables were created (see Table 3). Four control variables (17–20) were included to remove the effects of the design of products or services. The control variables were measures of a firm’s products or services: product or service (1–5), product/service differentiation (very similar–very different, 1–5), pricing (very cheap–very expensive, 1–5), and product/service quality (standard quality–very high quality, 1–5). Consequently, the control variables were used to determine how the different aspects of the products or services contributed to the firm’s growth. Therefore, the control variables were an isolating mechanism that allowed us to determine the firm’s dependences on sold products or services. In addition, the impact of the industry sector was initially assessed (manufacturing and knowledge-intensive sectors); however, no significant relationships with growth were found.

## Results and discussion

### Statistical analysis

An exploratory factor analysis with a principal component and a varimax rotation was used to determine the factors of entrepreneurial networks and proximity, and to analyze the validity of the hypothesized constructs. A factor loading exceeding 0.30 needs to be significant at the 0.05 level with a sample of 241 observations (Hair et al., 2006). There are also considerations regarding reliability, that is, the value of Cronbach’s alpha (α) (DeVillis, 1991; Bernstein and Nunnally, 1994). The lower limit of Cronbach’s alpha is 0.700 (Hair et al., 2006). However, in exploratory research, the lower limit can decrease to 0.600. George and Mallery (2003) state the following lower thresholds: “α > 0.900 – Excellent, α > 0.800 – Good, α > 0.700 – Acceptable, α > 0.600 – Questionable, α > 0.500 – Poor, and α < 0.500 – Unacceptable” (p. 231).

In this study, the factor analysis (see Table 4) generated three factors: geographical proximity (five variables, α = 0.779), professional network (six variables, α = 0.723), and consultative network (three variables, α = 0.444). However, the Cronbach alpha of the consultative network factor was too low and thus was dropped from our analysis. The Kaiser–Meyer–Olkin (KMO) value is > 0.600, and test statistics for Bartlett’s test of sphericity were 0.000. One variable, chamber of commerce, with a factor loading of 0.237, was dropped from further analysis because the factor loading was too low. Considering the statistical results, only two factors in the regression analysis were used.

**Table 4:** Factor analysis—Varimax axis factoring^a,b,c^—rotated factor matrix

Variables	Factor 1	Factor 2	Factor 3
Factor names	Geographical proximity	Professional network	Consultative network
Cronbach’s α	α = 0.779	α = 0.723	α = 0.444^d^
1. Accountants	0.089	0.035	**0.354**
2. Banking institutions	0.026	0.099	**0.744**
3. Chamber of commerce	0.023	0.078	0.237^e^
4. Consultants	0.211	0.293	**0.339**
5. Lawyers	0.065	**0.489**	0.234
6. Regional business partner	− 0.077	**0.633**	0.186
7. Patent advisers	− 0.096	**0.677**	0.065
8. Venture capital firms	− 0.038	**0.435**	0.032
9. Incubator network	0.032	**0.608**	0.016
10. Proximity to university	0.102	**0.494**	0.100
11. Proximity to customers	**0.622**	− 0.162	0.112
12. Proximity to competitors in the same field	**0.631**	− 0.002	0.109
13. Local/regional advantages	**0.367**	0.103	0.287
14. Attractive/expansive industrial area	**0.644**	0.101	0.028
15. Proximity to large, well-known firms	**0.661**	− 0.011	0.094
16. Proximity to similar firms	**0.719**	0.005	0.003

^a^Cumulative variance = 34.35%

^b^Cronbach α > 0.700, factor loading > 0.300

^c^KMO = 0.756 and Bartlett´s test of sphericity = 0.000

^d^Reliability too low. Excluded from further analysis

^e^Factor loading too low (< 0.300). Excluded from further analysis

The Pearson correlation analysis was also performed to predict initial factorability. Table 5 shows the correlations between geographical proximity, professional network, four control variables and initial growth. There is no correlation between geographical proximity and professional network. In Table 5, one of the four control variables (product/service) and one of the latent variables (professional network) are significant for firm growth. The correlations matrix determines the degree to which two variables or factors can move together, helps clarify the connection between two variables or factors, and provides information on the strength and direction of an association between variables or factors. The regression analysis in this study estimates parameters in a linear equation to predict values. In addition, Table B in the appendix shows that the statistical results at the variable level and the means are quite low, in terms of the level of interaction in entrepreneurial networks and proximity. The firms mainly produce services (mean 4.17: product-service), which is expected because 87 percent of the responding firms are high-tech knowledge-intensive firms. Product differentiation and pricing are in the middle (around three) and product quality is 3.91. Growth correlates with the variables of regional business partners, incubator networks, proximity to universities, attractive industrial areas, and the control variable, product or service, which is the only significant control variable (see Table B in the appendix).

**Table 5:** Correlation matrix

	Mean	Std	1	2	3	4	5	6
1. Geographical proximity	12.23	45.39						
2. Professional network	8.56	3.84	0.035					
3. Product or service	4.01	1.30	0.066	− 0.362**				
4. Product/service differentiation	3.00	1.25	− 0.148**	0.298**	–0.331**			
5. Pricing	2.81	0.91	0.047	0.028	0.073	− 0.66		
6. Product/service quality	3.91	0.80	− 0.071	− 0.043	0.039	0.102	0.363**	
7. Growth 2014–2016	3.41	15.32	0.068	0.216**	− 0.186**	0.030	− 0.070	0.005

*p < 0.05, **p < 0.01, ***p < 0.005.

Table 6 presents the six regression models, which is the third step in the statistical analysis. The regression analysis is based on the two latent variables that have acceptable reliability, which are the aggregated statistical means of the underlying variables. Models 1–3 show the regression analyses without control variables including the factors’ geographical proximity and professional networks. Models 4–6 show the regression analyses that include the four control variables. The six regression models assess the connections between the dependent variable growth between 2014 and 2016 and the independent variables. The latent variable of professional networks is significant in the models and has a positive impact. However, only one control variable significantly affects the relationship with growth (see Model 4). The factor for the professional network includes variables for lawyers, regional business partners, patent advisers, venture capital firms, incubator networks, and proximity to universities. The other factor, geographical proximity, is not significant in any of the models. The models that include the professional networks factor are all significant. However, the adjusted R squares are low. A statistical test was conducted to confirm our empirical findings. Multicollinearity can generate problems in regression analysis, and there is no indication of multicollinearity.

**Table 6:** Regression analysis

	Full sample *n* = 241			Full sample with control variables		
	Model 1	Model 2^a^	Model 3^a^	Model 4	Model 5^b^	Model 6^b^
Geographical proximity	0.016 (0.016)		0.016 (0.016)	0.015 (0.016)		0.012 (0.016)
Professional network		0.070*** (0.022)	0.070*** (0.022)		0.057* (0.024)	0.055* (0.024)
Product or service				− 0.168* (0.067)	− 0.107 (0.072)	− 0.110 (0.073)
Product/service differentiation				0.015 (0.073)	− 0.032 (0.074)	− 0.026 (0.074)
Pricing				− 0.095 (0.100)	− 0.127 (0.100)	− 0.128 (0.101)
Product/service quality				0.044 (0.115)	0.084 (0.114)	0.080 (0.115)
Constant	− 0.065 (0.217)	− 0.472* (0.203)	− 0.663 (0.282)	0.713 (0.606)	0.232 (0.639)	0.111 (0.663)
Adjusted R square	0.000	0.042	0.042	0.019	0.042	0.039

Dependent variable: firm growth 2014–2016; unstandardised coefficient betas and standard errors (between parentheses)

^a^The model is signifcant on the ***-level

^b^The model is significant on the *-level

*p < 0.05, **p < 0.01, ***p < 0.005

## Discussion

The relationships presented in this study provide an understanding of how network structures and geographical proximity affect NTBFs’ early growth. The resource-based perspective and network theory find that formal links support the development of firms and the ongoing existence of cooperative competencies, such as formal entrepreneurial networks. The findings, according to the empirical data, suggest that the creation of professional networks, including proximity to universities, is important for early sales growth. Furthermore, associations organized around industries (such as regional business partners and chambers of commerce in our study) is a form of formal networking. Informal networks are likely to be created at the earlier stages of a firm’s business cycle. Informal networks start with entrepreneurs’ utilizing their relationships with friends and family. Such informal networks can provide useful information or contacts that may be helpful in an NTBFs development. Informal ties (family members, and colleagues, etc.), which have been stated in other studies (Delmar & Shane, 2003; Hite & Hesterly, 2001; Sabbado et al., 2021), may be more dominant in the start-up stage of the firm. However, the informal aspects of such networks often mean they are taken for granted because the value as a network is not appreciated and may not be fully utilized. In this study, we selected organizations which are organized around the NTBFs as formal networks. An NTBF can acquire legitimacy by joining or cooperating with these organizations even before the firm has gained enough traction to become competitive. Entrepreneurial networks are often an effective way of reaching out to the public and regulators.

The theory of networks has gained traction over the years, with network research conceptualizing social structure as an enduring pattern of relationships among actors—individuals, groups, or organizations. The structure of network connections provides both opportunities and constraints; the network is difficult to imitate (Grant, 1991; Gulati, 1999). Some barriers hinder the imitation of resources (Lippman & Rumelt, 1982) and economies of scale (Rumelt, 1984, 1987). One possible explanation is that NTBFs are mainly located within industrial districts or science parks, where the geographical proximity between firms and other business actors could be regarded as important for the firms’ ability to develop networks and generate the resources needed to perform.

Several studies on industrial marketing management emphasize the importance of networks for newly established firms (Aaboen et al., 2013; Aaboen et al., 2016; Lassalle et al., 2020; Shih & Aaboen, 2017). Bhattacharyya and Ahmad (2010) state that apart from the classic economic theory, an efficient use of a network may create business or efficient business processes. Scholars find that different aspects of networks can be successful in a firm’s environment (Chu & Yoon, 2021; Gulati et al., 2000; Hite & Hesterly, 2001; Johannisson et al., 1994;). Entrepreneurs can shape their network structures using networking strategies (Engel et al., 2017). Therefore, over the years, entrepreneurial networks are deemed to be of significant importance (Barroso-Méndez et al., 2015).

**Fig. 2:**
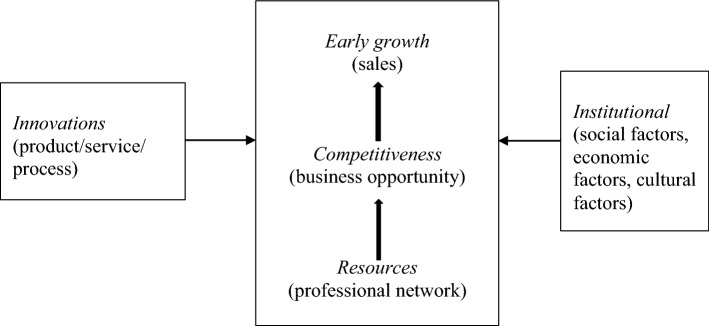
Schematic model: Key dynamics

Figure 2 illustrates the key dynamics and the need for network resources to achieve *competitiveness* (business opportunity) for NTBFs to attain early growth. A new entrepreneur can acquire business opportunities by joining a professional network. However, participation within the network is not without constraints, and the value that the NTBF can obtain from a professional network can change over time, consequently causing the network structure to change. The venture that is ready to enter the later-growth stage is in a more stable position (Chu & Yoon, 2021). Penrose (1959) claimed that differences in resources should be utilized to cause differences in competitive advantages. Arguably, networks are also found under specific conditions that support learning and joint knowledge creation, thereby creating a basis for competitive advantages. The NTBFs in forthcoming stages will also require a more comprehensive scope of resources than before. NTBFs at the initial stage may concentrate on acquiring business opportunities by constructing their professional networks. In later-growth stages, the NTBF may have to construct another type of network and various types of resources.

The statistical results indicate that the relationships are significant, and the professional network latent variable has a relationship with early growth. The professional network latent variable in our study comprises six variables: lawyers, regional business partners, patent advisers, venture capital firms, incubator networks, and proximity to universities. This latent variable is significantly related to growth, which indicates that in the initial stages, young firms should utilize professional networks to grow. The variable proximity to university is particularly interesting and was grouped in the factor professional network. Proximity is identified by scholars as one of the conditions for developing a network. According to the correlation matrix (Table B in the appendix), this single variable proximity to university has strong significant connections to consultants, lawyers, regional business partners, patent advisors, venture capital firms, incubator networks and to early firm growth. The variable proximity to university is hence the only single proximity variable that is significantly related to early growth.

The emergence of network ties evolves around restrictions such as proximity, industry, legal aspects, and socioeconomic factors such as personal knowledge and trust. However, environments in which firm processes can take place are expected to become more integrated over time. Growth, which is a time-related effect, may cause problems when an NTBF changes from a simple start-up to a slightly more complex firm, which requires new organizational efforts such as specialization of firm operations, markets, technology, and innovation and causes structural holes that have an impact on performance (Gulati et al., 2000). However, Freel (1998) and Teece et al. (1997) find that through path-dependent perspectives and accumulated learning processes, small entrepreneurial firms will expand their networks over time. Garnsey (1996) called this process “growth reinforcement,” where the firm-specific structural holes caused by growth are complemented by new networks necessary for the firm’s continuous development.

## Conclusions

In this study, we address entrepreneurship and performance from a networking perspective and analyze firms’ entrepreneurial networks and geographical proximity in relation to initial business performance, that is, NTBFs’ growth. The framework builds on contributions from the resource-based and networking theories, and a firm’s technology perspective. It is well known in management research that the resource-based theory focuses on business performance. The ability of NTBFs to mobilize resources and discover business opportunities is crucial for entrepreneurial networks because formal links mediate transactions. This study develops a model for early growth and entrepreneurial networks and geographical proximity and finds that the latent variable for professional networks is positively significant for NTBFs’ growth during their initial 3 years. Entrepreneurs therefore need to be proactive about professional networks and develop relationships that constantly evolve and establish formal links between NTBFs and business partners such as lawyers, venture capital firms, incubators, and universities.

Our study has practical implications and relevance for NTBFs, other individuals, or organizations such as regional business partners, incubators, and universities. Policy makers in this area normally try to develop entrepreneurial networks within specific population groups such as young or inexperienced entrepreneurs in locations including, for example, incubators. In this study, incubation networks represent a significant variable for early firm growth. Policy incentives can be mixed, that is, both new and experienced entrepreneurs can participate together in special programs that can offer both advice and encouragement.

To conclude, it seems that several formal links, such as regional business partners, incubator networks, and links to universities, positively affect growth. For example, an NTBF can acquire legitimacy if the firm belongs to an incubator program. However, only 10 percent of the 241 NTBFs in this study participated in an incubator program. In general, incubators’ business models include selection, business support, and mediation. The development of new high-tech firms is often of regional interest, and the roles of centers and support structures are aligned with regional development policies. Many regional areas attempt to improve their creative infrastructure knowledge centers, science parks, incubators, and regional business partners. These centers provide a geographical concentration of universities and research centers, which may transfer research results and commercialize them. Organizers of NTBFs, such as science parks and incubators, have to design programs for the initial stages of firm development, because entrepreneurial networks are important for business opportunities and resource allocations.

However, as in most studies, this study’s design had several limitations which can also be an agenda for future research on entrepreneurial networks, proximity, and growth. The questionnaire data is based on a single year, and entrepreneurial networks developed during the interaction processes. Growth is only measured across 3 years, and only the surviving NTBFs that started in 2013 were included in the analysis. This study therefore has a restricted empirical setting and therefore the results must be carefully interpreted. Future research could analyze the geographical proximity and entrepreneurial networks over a longer and examine how the networks emerge over time. Another limitation is that the growth potential for some NTBFs and the variables that are related to the founders’ backgrounds, such as education and business experience, can affect their entrepreneurial networks. Naturally, growth is more likely for very small and young firms. However, these firms are also likely to be at a disadvantage when it comes to expanding their networks.

This study focuses on industrial sectors characterized by dynamic business environments and high uncertainty, which in turn necessitates information gathering. Many studies in networking focus on economic development, with fewer studies considering other aspects such as sociological factors. Several studies have focused on how entrepreneurial networks help an entrepreneur to manage uncertainty and gain resources and market innovation opportunities. The theories used are normally based on the resource-based view, transaction costs, or social network aspects; moreover, many studies in entrepreneurship and management focus on economic issues rather than, for example, sociological issues to help explain entrepreneurial networks from a sociological point of view. Theories from a sociological viewpoint may further elucidate the structure of NTBF networking.

The data is for a period before the COVID-19 crisis, and it is difficult to determine the ways in which international and national crises impact firms’ networks and localizations because the regional and local impact of COVID-19 is probably heterogeneous. A general trend in the post-COVID society has been an increase in remote working and reliance on contingent workers, which creates both challenges and opportunities related to organizational proximity (proximity among people within an organization). Therefore, organizational proximity presents several interesting avenues for future research, including for example, how differences in organizational proximity affect the development of new entrepreneurial firms.

## Appendix

See Tables

**Table 7:** NACE Rev.2—sectors (responding firms)

*Sweden*	Sectors—frequencies (%)
Manufacture of chemicals and chemical products	0.4
Manufacture of fabricated metal products, except machinery and equipment	0.4
Manufacture of computer, electronic and optical products	2.6
Manufacture of electrical equipment	0.8
Manufacture of machinery and equipment n.e.c	3.7
Manufacture of motor vehicles, trailers and semi-trailers	0.8
Other manufacturing	0.8
Wholesale of mining, construction and civil engineering machinery	0.4
Motion picture, video and television programme production, sound recording	11.6
Telecommunications	2.1
Computer programming, consultancy and related activities	58.1
Information service activities	7.1
Activities of head offices; management consultancy activities	0.4
Architectural and engineering activities; technical testing and analysis	2.9
Scientific research and development	7.1
Other professional, scientific and technical activities	0.8 100.0

^7^ and

**Table 8:** Correlation matrix on the variable level—entrepreneurial networks and geographical proximity

Variables		1	2	3	4	5	6	7	8	9	10	11	12	13	14	15	16	17	18	19	20
1	Accountants																				
2	Banking institutions	0.266**																			
3	Chamber of commerce	0.159*	0.143*																		
4	Consultants	0.100	0.300**	0.203**																	
5	Lawyers	0.115	0.202**	0.164*	0.195**																
6	Regional business partner	0.103	0.193**	0.054	0.227**	0.288**															
7	Patent advisers	0.024	0.136**	0.047	0.199**	0.364**	0.464**														
8	Venture capital firms	− 0.029	0.113	0.007	0.130*	0.300**	0.233**	0.326**													
9	Incubator network	0.054	0.047	0.103	0.199**	0.261**	0.429**	0.373**	0.264**												
10	Proximity to university	0.119	0.126	− 0.016	0.153*	0.259**	0.370**	0.317**	0.197**	0.318**											
11	Proximity to customers	0.107	0.096	− 0.027	0.080	− 0.030	− 0.096	− 0.185**	− 0.069	− 0.111	0.016										
12	Proximity to competitors in the same field	0.092	0.081	0.071	0.260**	0.078	− 0.042	− 0.097	− 0.030	0.038	0.075	0.338**									
13	Local/regional advantages	0.122	0.291**	0.019	0.150*	0.119	0.138*	− 0.077	−0.031	0.123	0.203**	0.230**	0.262**								
14	Attractive/expansive industrial area	0.040	0.041	0.053	0.136*	0.127*	− 0.032	0.033	0.009	0.094	0.155*	0.445**	0.317**	0.330**							
15	Proximity to large, well-known firms	0.142*	0.098	0.032	0.150*	0.066	− 0.057	0.077	0.033	− 0.053	− 0.026	0.489**	0.363**	0.242**	0.521**						
16	Proximity to similar firms	0.161	0.013	.024	0.214**	0.037	− 0.047	− 0.115	− 0.035	0.076	0.099	0.385**	0.625**	0.249**	0.372**	0.445**					
17	Product or service	0.086	− 0.003	0.005	− 0.065	− 0.103	− 0.359**	− 0.338**	− 0.272**	− 0.104	− 0.253**	0.144*	0.056	− 0.079	− 0.004	0.048	0.094				
18	Product/service differentiation	0.043	0.028	0.062	0.012	0.171**	0.209**	0.196**	0.215**	0.189**	0.196**	− 0.102	− 0.120	− 0.050	− 0.075	− 0.123	− 0.156*	− 0.331**			
19	Pricing	0.005	0.027	0.005	0.044	0.087	− 0.025	0.047	− 0.035	0.083	− 0.037	0.005	0.087	− 0.012	− 0.029	0.128	0.024	0.073	− 0.066		
20	Product/service quality	0.029	− 0.001	− 0.002	0.082	0.065	− 0.066	− 0.015	0.000	− 0.078	− 0.036	0.010	0.052	− 0.001	0.031	0.113	0.099	− 0.039	0.102	0.363**	
21	Growth 2014–2016	− 0.079	0.007	0.049	0.098	0.121	0.179**	0.1030	0.083	0.181**	0.153*	− 0.026	0.004	0.099	0.155*	0.039	0.004	− 0.186**	0.030	− 0.070	0.005

**p* < 0.05, ***p* < 0.01, ****p* < 0.005

^8^.
